# Investigating increased admissions to neonatal intensive care in England between 1995 and 2006: data linkage study using Hospital Episode Statistics

**DOI:** 10.1186/s12874-016-0152-0

**Published:** 2016-05-20

**Authors:** Andrei S. Morgan, Neil Marlow, Kate Costeloe, Elizabeth S. Draper

**Affiliations:** Institute for Womens’ Health, UCL, 74 Huntley Street, London, UK; Homerton Hospital, London, UK; University of Leicester, Leicester, UK

**Keywords:** Extreme prematurity, Record linkage, Hospital Episode Statistics, England

## Abstract

**Background:**

A 44 % increase was observed in admissions to neonatal intensive care of babies born ≤26 weeks completed gestational age in England between 1995 and 2006. Hospital Episode Statistics (HES) may provide supplementary information to investigate this. The methods and results of a probabilistic data linkage exercise are reported.

**Methods:**

Two data sets were linked for each year (1995 and 2006) using 3 different algorithms (Fellegi and Sunter, Contiero and estimation-maximisation).

**Results:**

In 1995, linkage was performed between 668 EPICure and 486,705 HES records; 1,820 linked pairs were identified of which 422 (63.17 %) were confirmed. In 2006, from 2,750 EPICure and 631,401 HES records, 8,913 linked pairs were identified with 1,662 (60.40 %) confirmed as true. Reported births in HES at <26 weeks gestation increased 37.0 % from 867 to 1188.

**Conclusions:**

Results support the EPICure findings that there was an increase in the birth rate for extremely premature babies between 1995 and 2006. There were insufficient data available for detailed investigation. Routine data sources may not be suitable for investigations at the margins of viability.

**Electronic supplementary material:**

The online version of this article (doi:10.1186/s12874-016-0152-0) contains supplementary material, which is available to authorized users.

## Background

Survival of extremely premature babies has improved in recent decades [1], particularly following the introduction of antenatal steroids and postnatal surfactant [2–4]. Previous work suggests that routine data sources collected in England such as Hospital Episode Statistics (HES) or the Births and Deaths Registry provide insufficient data to permit detailed investigation [5, 6].

Between March and December 1995, the EPICure study collected information on all babies born in Great Britain and the Republic of Ireland at less than 26 weeks completed gestation who were admitted into neonatal intensive care [7]. The EPICure 2 study collected data on all births occurring below 27 weeks gestation in England during the whole of 2006 [8]. Comparison of the two data sets demonstrated a disproportionate 44.0 % increase in the number of babies admitted into intensive care at less than 26 weeks [8], whereas the birth rate overall increased by only 0.7 %, from 613,257 to 635,748 per year [9].

It is unclear whether the increase seen represents a true rise in the numbers of live babies being born extremely prematurely or if, instead, it reflects changes in management in the delivery room. To investigate this question, we required specific extra demographic data for 1995 to explain the rise in admission rates. We therefore sought to supplement the EPICure data sets with additional information from Hospital Episode Statistics by performing probabilistic data linkage between each of the two data sets (EPICure and HES) available for 1995 and 2006 as there were insufficient patient identifying variables to permit deterministic linkage.

## Methods

### Available data sets

In 1995, EPICure collected a brief delivery room log comprising date of birth, gestational age, birth weight and infant sex; a more complete data set was only collected for babies admitted into neonatal intensive care. For the EPICure 2 study, delivery data were collected using an expanded form which comprised extensive delivery and resuscitation data with the help of the Confidential Enquiry into Maternal and Child Health (CEMACH). HES data for 1995 and 2006 were obtained from the NHS Health and Social Care Information Centre (HSCIC); the full list of the variables requested and the available data that were returned is shown in Table 1 of Additional file 1.

**Table 1:** Probability estimates for linkage analyses

Matching variable	Baseline best guesses				Dattani et al. [15] estimate			
	*m*	*u*	$w_{\mathrm {m}}^{\mathrm {a}}$	$w_{\text {nm}}^{\mathrm {b}}$	*m*	*u*	$w_{\mathrm {m}}^{\mathrm {a}}$	$w_{\text {nm}}^{\mathrm {b}}$
Date of birth	0.90	0.00274	5.794	-2.3	0.7405	0.0015	6.202	-1.347
GA at birth	0.80	0.02	3.689	-1.589	0.4941	0.0494	2.3028	-0.6308
Sex	0.999	0.49	0.7123	-6.2344	0.7208	0.0062	4.756	-1.270
Discharge date	0.20	0.002	4.6052	-0.2211	—	—	—	—
Date of death ^c^	0.20	0.00274	4.2904	-0.2204	0.30	0.002	5.0106	-0.3547
Birth weight	0.60	0.001	6.3969	-0.9153	0.7405	0.0074	4.606	-1.342
Birth order	0.87	0.95	-0.08797	0.95551	0.8153	0.0033	5.510	-1.686
Delivery method ^c^	0.80	0.80	0	0	0.67	0.1	1.902	-1.003
Ethnic category	0.20	0.10	0.6931	-0.1178	0.7308	0.095	2.040	-1.212
Mother’s age at delivery	0.95	0.05	2.944	-2.944	—	—	—	—
Mother’s date of birth	0.90	0.0001	9.105	-2.302	—	—	—	—
Postcode	0.90	0.001	6.802	-2.302	0.9291	0.065	2.660	-2.579
Number of previous pregnancies	0.60	0.90	-0.4055	1.3863	—	—	—	—
Number of babies	0.95	0.95	0	0	0.8153	0.0033	5.510	-1.686

Probability estimates for linkage analyses between Hospital Episode Statistics and EPICure data based on best guesses and prior knowledge (adapted from data linkage performed by Dattani et al between Hospital Episode Statistics (HES) and NHS Numbers 4 Babies data sets) [15]

^a^ *w*_m_= weight if pairs match

^b^ *w*_nm_= weight if pairs do not match

^c^Date of death and delivery method were both modified using an adjusted best guess for the second linkage analysis performed using estimates from Dattani et al.

Each data set was cleaned, then restricted to variables required for matching. A detailed explanation of how the HES and EPICure data sets potentially match is included in Additional file 2. All analyses were conducted using R [10].

### Choice of variables

Variables chosen for inclusion in the matching exercise were: baby’s date of birth, sex, gestational age and weight at birth, birth order, total number of babies in the pregnancy, the mother’s number of previous pregnancies, discharge date, maternal age at delivery, date of death, ethnicity and postcode. Maternal age at delivery was included in preference to maternal date of birth to minimise errors from data entry; date of death was derived for HES using “date of discharge” and “discharge method”. “Ethnic category” was recoded to match the EPICure categorisation and was included even though supplementary information on ethnicity was one of the desired results. Derived variables and ethnicity were included in the matching for 1995 to improve subject discrimination as postcode was unavailable.

### Linkage criteria

Linkage was performed for both study epochs in the same way. Each of three algorithms available in the “RecordLinkage” package [11] of R [10] were used. These are based on the estimation-maximisation algorithm [12] and on the methods of Fellegi & Sunter (stochastic linkage) [13], and Contiero (EpiLink algorithm) [14]. For the Fellegi & Sunter analysis, weights (*w*) are calculated stochasticly, based on *M* (i.e. that both records of a pair are from the same subject) and *U* (where records in a pair belong to different subjects) probabilities [11]. We performed one round of matching using “best guess” values, and a second round using estimates from Dattani et al. [15], supplemented where no prior information was available with the “best guess” estimates. Values are shown in Table 1. A more detailed explanation of the linkage methods is provided in Additional file 3.

### Sensitivity analyses

Direct comparison (i.e. using different parameters) was made between the two versions of the stochastic linkage using the “best guess” and Dattani probabilities.

### Thresholds

Data were tabulated to identify appropriate cut off points for clerical review. Initial thresholds attempted only to obtain a “reasonable” number of matches for review; subsequent revision was not possible due to time limitations.

### Clerical review

Following linkage, a master data set was created for each epoch by combining data for retained ID pairs. Rows corresponding to duplicate entries of a single EPICure ID were manually reviewed. For true matches, that specific row and all other potential matches with those IDs were removed from further consideration. The review process was repeated using both the EPICure and HES subjects as the base for comparison until no further true matches could be identified.

### Error measures

**Fig. 1:**
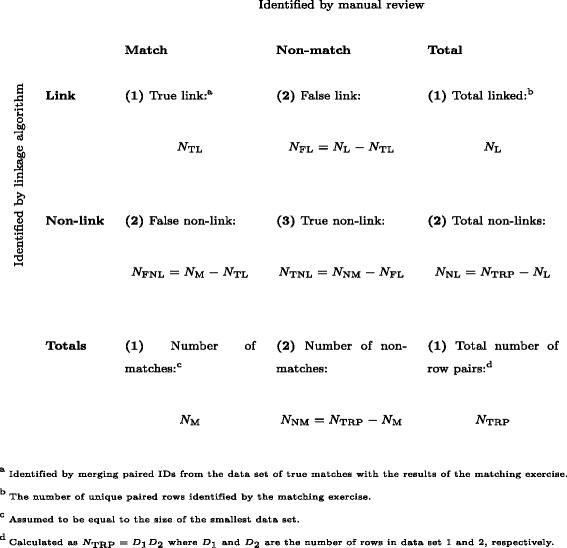
Known and calculated values for matching algorithms, used in assessment of linkage error. Data linkage is performed by pairing data from two data sets, followed by manual verification of linked pairs to identify true matches. Values for cells were identified in the following manner: (1) The total number of row pairs, maximum number of matches, total number of linked pairs and number of true matches within those linked pairs were identified. (2) The numbers of false links, false non-links, total non-links and number of non-matches were then derived. (3) Finally, the true number of non-matches among the non-linked pairs was calculated

For each method of linkage, we assessed matching accuracy by merging the true matches with the saved unique pairs. This permitted the number of “true matches” to be identified and enabled sensitivity, specificity, positive predictive value and negative predictive value to be calculated (see Fig. 1).

## Results

For the 1995 EPICure cohort, data from 668 babies who were admitted into neonatal intensive care were available. The EPICure 2 data set contained 4,144 rows which, after removal of data not collected in HES (terminations of pregnancy: 768; still births: 626), resulted in 2,750 individual subject records being available.

Hospital Episode Statistics (HES) data were supplied by the NHS HSCIC for each year of analysis. There were 575,509 records for 1995 and 631,499 for 2006. To match the time period of the EPICure study, births occurring in January or February were excluded from the 1995 data; 8,807 records with a missing date of birth were retained, meaning 486,705 records were used for linkage. There were no duplicates in 1995; in 2006, 98 duplicate rows were removed, leaving 631,401 records for analysis.

### Data quality

Postcode and, consequently, Socioeconomic data were completely absent from HES data for 1995, and fewer than 20.0 % of the subjects had information on ethnicity. For 2006, socioeconomic information was available for over 50.0 % of subjects, and ethnicity unavailable for only 157,781 (25.0 %) records. Levels of *missingness* for matching variables are shown in Table 2: HES data were less complete for each time period. Expanded distributions for gestational age and birth month are shown in Additional file 4: Table S1.

**Table 2:** *Missingness* among the matching variables

Variable	HES 1995	EPICure (1995)	HES 2006	EPICure-2 (2006)
	Missing (%)	Missing (%)	Missing (%)	Missing (%)
Date of birth	8807 (1.53)	0 (0.00)	4265 (0.68)	0 (0.00)
GA at birth	164006 (28.50)	0 (0.00)	336178 (53.23)	7 (0.25)
Sex	2616 (0.45)	0 (0.00)	3202 (0.51)	9 (0.33)
Discharge date	16912 (2.94)	373 (55.84)	—	—
Date of death	571417 (99.29)	268 (40.12)	—	—
Birth weight	152641 (26.52)	0 (0.00)	288014 (45.61)	26 (0.95)
Birth order	250718 (43.56)	0 (0.00)	224632 (35.57)	0 (0.00)
Delivery method	168018 (29.19)	1 (0.15)	—	—
Number.of babies	152378 (26.48)	0 (0.00)	209455 (33.17)	0 (0.00)
Previous pregnancies number	—	—	618692 (97.97)	101 (3.67)
Ethnic category	462999 (80.45)	0 (0.00)	—	—
Postcode	—	—	290462 (46.00)	1 (0.04)
Mother’s dob	—	—	273426 (43.30)	2750 (100.00)
Mother’s age at delivery	214999 (37.36)	4 (0.60)	273430 (43.30)	8 (0.29)

Variables in each of the Hospital Episode Statistics and EPICure data sets that were used for matching in 1995 and 2006 and their levels of *missingness*. (HES (1995) *n*=575,509 (for the entire year); EPICure *n*=668 (March – December); EPICure 2 *n*=2,750; HES (2006) *n*=631,401)

#### Data concordance

In 1995, 2,184 (82.9 %) of 2,634 subjects in HES with a recorded birth weight of less than 500 grams were described as having a gestational age of 35-45 weeks (Table 3). For those recorded as being of a low gestational age, birth weight was missing in 14.3 %, 11.4 %, and 6.2-7.5 % at 20, 21 and 22 to 23 weeks gestational age respectively, and in only 0.2 % of those born between 35 and 39 weeks. In 2006, problems were even greater with the entire data set (Additional file 4: Table S2). No issues were identified with either of the EPICure data sets.

**Table 3:** Birth weight v gestational age in HES 1995 data

Birth weight category	Gestational age (weeks)											
	20	21	22	23	24	25	26-29	30-34	35-39	40+	Missing	Total
<500	19	23	39	36	53	40	117	91	960	1224	32	2634
	(0.003)	(0.004)	(0.007)	(0.006)	(0.009)	(0.007)	(0.020)	(0.016)	(0.167)	(0.213)	(0.006)	(0.458)
500-999	3	7	29	103	273	292	864	181	36	19	89	1896
	(0.001)	(0.001)	(0.005)	(0.018)	(0.047)	(0.051)	(0.150)	(0.031)	(0.006)	(0.003)	(0.015)	(0.329)
1000-1499	0	1	0	1	6	20	1179	1590	326	180	152	3455
	(0.000)	(0.000)	(0.000)	(0.000)	(0.001)	(0.003)	(0.205)	(0.276)	(0.057)	(0.031)	(0.026)	(0.600)
1500-1999	0	2	1	0	4	2	141	3785	2010	94	224	6263
	(0.000)	(0.000)	(0.000)	(0.000)	(0.001)	(0.000)	(0.025)	(0.658)	(0.349)	(0.016)	(0.039)	(1.088)
2000-2499	0	0	0	1	0	2	26	3367	13684	1831	617	19528
	(0.000)	(0.000)	(0.000)	(0.000)	(0.000)	(0.000)	(0.005)	(0.585)	(2.378)	(0.318)	(0.107)	(3.393)
2500-2999	0	0	1	0	0	5	24	1053	47425	21521	2069	72098
	(0.000)	(0.000)	(0.000)	(0.000)	(0.000)	(0.001)	(0.004)	(0.183)	(8.241)	(3.739)	(0.360)	(12.528)
3000-3499	0	5	2	3	6	2	30	334	67091	80740	4400	152613
	(0.000)	(0.001)	(0.000)	(0.001)	(0.001)	(0.000)	(0.005)	(0.058)	(11.658)	(14.029)	(0.765)	(26.518)
3500-3999	2	1	4	1	3	5	13	117	33709	82270	3480	119605
	(0.000)	(0.000)	(0.001)	(0.000)	(0.001)	(0.001)	(0.002)	(0.020)	(5.857)	(14.295)	(0.605)	(20.782)
4000-4499	0	0	0	0	0	0	5	32	7202	29943	1040	38222
	(0.000)	(0.000)	(0.000)	(0.000)	(0.000)	(0.000)	(0.001)	(0.006)	(1.251)	(5.203)	(0.181)	(6.641)
4500-4999	0	0	0	0	0	0	2	4	916	4763	175	5860
	(0.000)	(0.000)	(0.000)	(0.000)	(0.000)	(0.000)	(0.000)	(0.001)	(0.159)	(0.828)	(0.030)	(1.018)
5000+	0	0	0	1	1	0	8	4	132	520	28	694
	(0.000)	(0.000)	(0.000)	(0.000)	(0.000)	(0.000)	(0.001)	(0.001)	(0.023)	(0.090)	(0.005)	(0.121)
Missing	4	5	6	10	23	30	110	169	315	269	151700	152641
	(0.001)	(0.001)	(0.001)	(0.002)	(0.004)	(0.005)	(0.019)	(0.029)	(0.055)	(0.047)	(26.359)	(26.523)
Total	28	44	82	156	369	398	2519	10727	173806	223374	164006	575509
	(0.005)	(0.008)	(0.014)	(0.027)	(0.064)	(0.069)	(0.438)	(1.864)	(30.200)	(38.813)	(28.498)	(100.000)

Numbers of subjects (percentages of overall data set) according to birth weight (g) by gestational age (weeks), as recorded in the 1995 Hospital Episode Statistics data set

#### Stochastic analysis - baseline estimated values

**Fig. 2:**
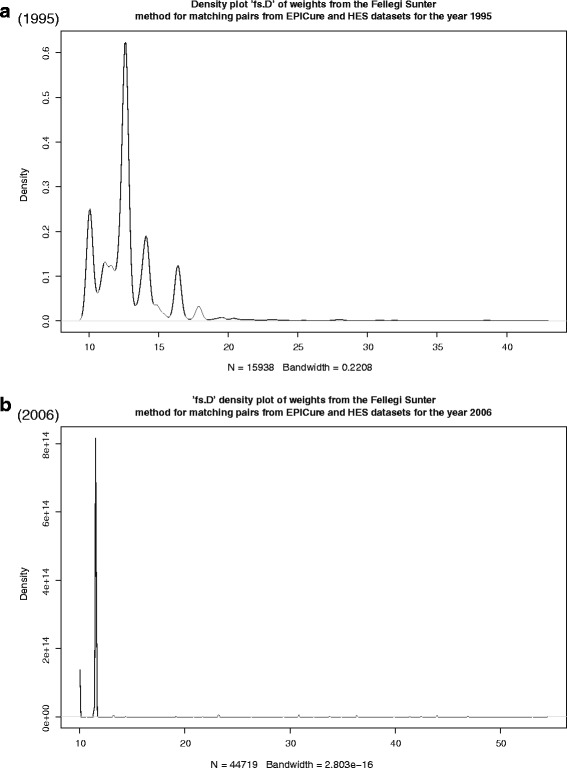
Density distribution of weights from the stochastic linkage analyses using best guess probabilities. Axes are not to the same scale

**Fig. 3:**
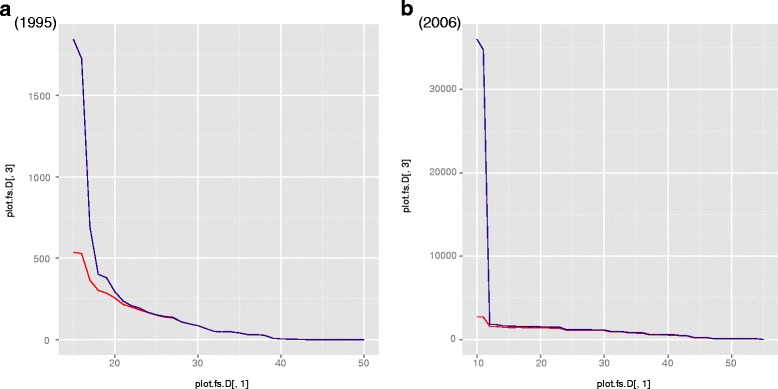
Numbers of individual matches according to weight from each of the Hospital Episode Statistics (HES) (*blue line*) and EPICure (*red line*) data sets in the stochastic linkage analysis using best guess probabilities. “Weight” is on the *x*-axis, number of matches on the *y*-axis; axes are not to the same scale

In 1995, the maximum weight of a linked pair was 42.3. 2,093 unique pairs were identified above a threshold of 15, representing 537 EPICure IDs and 1,846 HES IDs. There was a marked drop above 17, to 792 unique pairs (365 unique EPICure IDs and 692 unique HES IDs, Table 4). This is seen in the density graph of weights (coded “fs.D” in Fig. 2a), and in the number of unique records linked from each data set (Fig. 3a). Above 30, the number of linked pairs equalled the number of IDs from each data set – i.e. there were 86 uniquely matched pairs.

**Table 4:** Number of pairs matched using guestimate probabilities (1995)

Cut off weight	N pairs	N EPICure	% EPICure	N HES	% HES
15	2093	537	80.39	1846	0.38
16	1939	528	79.04	1726	0.35
17	792	365	54.64	692	0.14
18	467	302	45.21	401	0.08
19	435	285	42.66	380	0.08
20	335	256	38.32	294	0.06
21	270	216	32.34	237	0.05
22	229	200	29.94	208	0.04
23	202	182	27.25	193	0.04
24	175	166	24.85	167	0.03
25	158	150	22.46	152	0.03
26	145	138	20.66	142	0.03
27	140	133	19.91	137	0.03
28	112	110	16.47	109	0.02
29	97	96	14.37	96	0.02
30	86	86	12.87	86	0.02
31	67	67	10.03	67	0.01
32	50	50	7.49	50	0.01
34	47	47	7.04	47	0.01
35	41	41	6.14	41	0.01
37	31	31	4.64	31	0.01
38	26	26	3.89	26	0.01
39	9	9	1.35	9	0.00
40	4	4	0.60	4	0.00
42	2	2	0.30	2	0.00
43	0	0	0.00	0	0.00

Table of the number of pairs in 1995 matched from each data set for differing cutoffs in the value of the weight calculated by the Fellegi-Sunter (guestimate) method of data linkage

The maximum weight in 2006 was 54.51; a cut-off of 10 was chosen. Graphs for 2006 are shown in Figs. 2b (density graph) and 3b (unique IDs). Above the cut-off value, there were 44,719 unique record pairs identified, representing 2,729 unique EPICure 2 IDs and 36,025 HES IDs. A large decrease was seen above a cut-off of 12, to 2,459 pairs overall with 1,569 and 1,811 unique EPICure 2 and HES IDs, respectively (Additional file 4: Table S3).

#### Stochastic analysis - Dattani estimates

With the Dattani et al. [15] probabilities, the maximum weight in 1995 was 65.7 and, in 2006, 71.57; thresholds of 35 and 15 were chosen, respectively. In 2006, there were 53,413 potential links with the number of HES IDs dropping from 32,051 to 3,129 between weights of 18 and 19. In 1995, there was a relatively constant decrease in the number of EPICure IDs, whereas the number of potentially linked HES IDs dropped from 16,385 to 3,540 at a weight of 36. Full details are presented in Additional file 4: Table S4 (for 1995) and Additional file 4: Table S5 (for 2006), with graphs shown in Additional file 5: Figure S1 (density graphs) and Additional file 5: Figure S2 (unique IDs linked).

#### Contiero algorithm

For both years, a cut-off value of 0.35 was chosen. 45,349 pairs were retained in 1995 compared to 6,323 in 2006. There was a much better spread of weights in 2006 (see density graphs, Additional file 5: Figure S3), reflected by the maximum weight obtained in each of the analyses: 0.9494 in 2006 but only 0.8678 in 1995.

Convergence in the numbers of matched IDs occurred around a weight of 0.45 in both epochs. Unique matches were only identified in 1995 above a threshold of 0.75 (20 pairs – Table 5), with none identified in 2006 (Additional file 4: Table S6). Graphs of unique IDs from each data set are shown in Additional file 5: Figure S4.

**Table 5:** Number of pairs matched using the EpiLink algorithm (1995)

Cut off weight	N pairs	N EPICure	% EPICure	N HES	% HES
0.35	45349	662	99.10	38163	7.84
0.40	9329	612	91.62	8533	1.75
0.45	1670	421	63.02	1541	0.32
0.50	492	279	41.77	461	0.09
0.55	213	193	28.89	209	0.04
0.60	157	147	22.01	153	0.03
0.65	117	111	16.62	114	0.02
0.70	78	74	11.08	78	0.02
0.75	51	51	7.63	51	0.01
0.80	20	20	2.99	20	0.00
0.85	8	8	1.20	8	0.00
0.90	0	0	0.00	0	0.00
0.95	0	0	0.00	0	0.00
1.00	0	0	0.00	0	0.00

Table of the number of pairs matched in 1995 from each data set for differing cutoffs in the value of the weight calculated by the EpiLink (Contiero) method of data linkage

#### Estimation-maximisation likelihood algorithm

For the estimation-maximisation algorithm, the maximum weight in 1995 was 65.7, and 71.57 in 2006 with a threshold of 10 used for both analyses. There was a steadier attenuation in the number of linked pairs in 1995 than 2006 (Additional file 5: Figure S5a and S5b show the unique IDs; density graphs are shown in Additional file 5: Figure S6). In 1995, only above a weight of 43 were pairs uniquely matched (Additional file 4: Table S7), and in 2006, only two unique pairs were identified – above a weight of 70 (Additional file 4: Table S8).

### Manual review of linked pairs

1,820 linked pairs from the different analyses in 1995, and 8,913 in 2006, were concatenated together to create data sets of unique pairs – 1,070 in 1995 and 4,378 in 2006. 1995 data were manually reviewed four times, confirming 422 matches between the EPICure and HES data (63.2 % of the 668 maximum potential matches). For 2006, three rounds of manual review were performed, reducing the data set to 1670 rows which included 1,666 unique EPICure 2 and 1,670 unique HES IDs. Insufficient data were available to discriminate among the four remaining EPICure 2 IDs, each of which were paired with two HES IDs. Discarding these unconfirmed links meant that overall there were a total of 1,662 confirmed of a maximum 2,750 possible matches – 60.4 %.

### Assessment of error

We calculated sensitivity, specificity, positive and negative predictive values. In all analyses, specificity and negative predictive value were 1.0. The Fellegi-Sunter analysis using baseline best guesses provided the most accurate results in both epochs, correctly identifying 402 pairs in 1995, and 1740 in 2006. It also had the highest sensitivity in each time period – although it only identified 63.3 % of subjects in 2006, and 60.2 % in 1995. Results are presented in Tables 6 and 7 for 1995 and 2006, respectively.

**Table 6:** Linkage error measures (1995)

Linkage algorithm	Cutoff	True matches	PPV	Sensitivity
EM	10.00	238	0.005	0.356
EpiLink (Contiero)	0.35	387	0.009	0.579
FS (baseline model)	15.00	402	0.192	0.602
FS (Dattani estimates)	35.00	244	0.008	0.365

Positive predictive value (PPV) and sensitivity of results obtained using different methods for linkage between the HES and EPICure data sets in 1995. *EM*: estimation-maximisation, *FS*: Fellegi-Sunter

**Table 7:** Linkage error measures (1995)

Linkage algorithm	Cutoff	True matches	PPV	Sensitivity
EM	10	1408	0.025	0.512
EpiLink (Contiero)	0.35	1501	0.237	0.546
Fellegi-Sunter (baseline model)	10	1740	0.039	0.633
Fellegi-Sunter (Dattani estimates)	15	1665	0.031	0.606

Positive predictive value (PPV) and sensitivity of results obtained using different methods for linkage between the HES and EPICure data sets in 2006. *EM*: estimation-maximisation, *FS*: Fellegi-Sunter

### Saved HES data

During the 10 months of the EPICure study in 1995, from 1^st^ March to 31^st^ December, there were 867 births recorded in HES with a gestational age of 25 weeks or lower. These were merged with the 422 “true” matches identified in the probabilistic linkage; there were 300 matches, leaving 567 subjects for whom no further investigation was possible. In 2006, there were 2,535 HES records identified of births at less than 27 completed weeks gestational age. These were combined with the 1,662 records from the probabilistic matching: there were 932 matching rows, leaving 1603 for whom further review was not possible.

### Answering the original question

In advance of record linkage, it was realised the original question could not be answered due to lack of additional data. However, a cautious investigation of demographic shifts between 1995 and 2006 using the HES data alone was possible. Identical populations to EPICure could not be identified: HES records data from live births of less than 24 weeks and for *all* births at 24 weeks gestation and above, and does not distinguish live births who died in the delivery room from those who were admitted into neonatal intensive care but died on the same calendar date.

Table 8 shows how the data changed between the two study epochs. There were 867 births reported in HES in 1995 of <26 completed weeks gestational age; 213 were still births. Examining this in relation to the corresponding 2006 data (i.e. also of less than 26 completed weeks gestational age and from a similar time period) shows a 37.0 % increase in reported births. For reported live births, there was a 42.8 % increase. The table also contains data about three other populations from HES:

1. The “true” population: this contains data for HES subjects identified by the linkage exercise following clerical review.

**Table 8:** Changes in the number of births in HES data over time

HES data set ^a^	1995 ^b^				2006 (<26 weeks) ^c^				Percentage change ^d^	2006 (<27 weeks) ^e^			
	Live	Still	Not	Total	Live	Still	Not	Total		Live	Still	Not	Total
	births	births	known		births	births	known			births	births	known	
Reported	621	213	33	867	887	121	180	1188	37 %	1856	201	278	2535
“True”	396	16	10	422	699	127	187	1013	140 %	1158	213	291	1662
“Confirmed”	282	13	5	300	412	81	75	568	89 %	684	134	114	932
“Misclassified”	339	200	28	567	475	40	105	620	9 %	1172	67	364	1603

Changes in the number of births in Hospital Episode Statistics (HES) data between 1995 and 2006: reported, “true”, “confirmed” and “misclassified” data

^a^For each year, data sets were created based upon : a) gestational age as reported in the original HES data; b) only the “true” data identified by the data linkage exercise (i.e. contained in both HES and EPICure); c) HES data “confirmed” by the “true” data; and, d) “misclassified” data, which are those reported by HES but that were not identified as “true” during data linkage

^b^In 1995, data were available from March 1^st^ – December 31^st^ for babies of <26 completed weeks gestational age

^c^Comparison data sets from 2006 were created to include babies born between 1^st^ March and 31^st^ December at less than 26 weeks gestational age

^d^The total percentage increase in all births is presented

^e^The complete data sets from 2006 include births of <27 completed weeks gestational age from the entire year
2. The “confirmed” population: represents those reported in HES as below 26 (and, for 2006, also below 27) weeks who were confirmed by the linkage exercise.
3. The final group contains those from the reported group who were *not* identified during linkage.

## Discussion

We were unable to confirm the hypothesis that HES data are a suitable data source with which to investigate the apparent 44 % increase neonatal admissions between 22 and 25 completed weeks gestational age that was seen between 1995 and 2006. Overall, only approximately 60 % of available EPICure records were successfully linked in each study epoch using a combination of probabilistic methods. Of three linkage methods utilised, the Fellegi and Sunter technique using “best-guess” estimates of matching probabilities was the most successful in 1995, with no clear “best technique” in 2006.

Examination of the HES data demonstrated an increase of 37.0 % in the number of reported births between 1995 and 2006, and 42.8 % in live births in a population similar to that of the EPICure studies (less than 26 weeks gestational age and born between March 1^st^ and December 31^st^). This suggests the 44.0 % increase in admissions to neonatal intensive care seen in the EPICure data might be real. However, there were insufficient other data (ethnicity, socioeconomic status) to permit detailed investigation.

### Data considerations

Hospital Episode Statistics is a routine data set collected since 1989 from secondary care sources with primarily non-clinical motives [16]. Birth data in HES are incomplete. Births in non-NHS locations (private hospitals or birthing centres, or at residential locations) may not be collected, and there is marked variation in reporting by different health care providers [5,15,17]. Data may be entered by midwives immediately after delivery via point-of-care systems or separately by clinical coders; reporting practices have changed over time [17]. In contrast, the EPICure data were specific cohort studies run in collaboration with national confidential enquiries (Confidential Enquiry into Stillbirths and Deaths in Infancy (CESDI) and CEMACH) [7,8]. Data were *only* collected about specific births by those directly involved in care under the responsibility of a delegated EPICure contact (usually a doctor) at each perinatal centre in England [7,8].

These differences were apparent. The EPICure data are more likely to be accurate with respect to gestational age as these were rechecked against source data and recalculated if necessary [7,8]. In the HES data, there were inconsistencies between gestational age and birth weight category as well as deficiencies in data quantity. High levels of missing data were seen in variables used for linkage; many others contained a complete absence of data. This severely limited the capacity for accurate data linkage and prevented further meaningful investigations.

### Methodological considerations

HES data problems may have biased the results. Population coverage may have led to selection bias if data were less well reported in some regions or for some hospitals than in the EPICure studies, thus resulting in matches not being identified when they could have been. Information bias is likely as a consequence of HES data consistency issues. Similar work linking HES with maternity data for England and Wales has shown low rates of discordance between sources [15,18–22]; however, data quality issues are more likely to be an issue for those born in unusual circumstances like those who are extremely premature. Such errors are likely to apply across the gestational age ranges included in this study, thus causing non-differential misclassification and biasing linkage towards non-identification of true matches.

Confounding cannot be excluded, but is unlikely. It might occur if birth weight were closely correlated with gestational age – but this was not the case. No other matching variables in this study would be expected to show a strong correlation with extreme prematurity *and* successful linkage.

Random error may also affect analyses, but given that the purpose of probabilistic linkage is only to assign a weight, it is unlikely to be of great importance. This is because manual intervention is required, if not for review purposes, at least for selection of a threshold. This consequently provides a counterbalance to random error: an acceptable level of error is determined by the number of records to review.

A different problem arises from combining the results from the different analyses prior to manual review. This shortened analysis time, but introduced contamination between the different linkage methods. This is because remaining pairs with HES or EPICure IDs corresponding to those in an identified “true” match were removed, meaning identification of a match from one analysis potentially influenced the choice of match arising from another.

#### Fellegi and Sunter analysis

For the best guess analyses, *M* and *U* probabilities but not the resultant weights were considered in advance. However, where the *M* and *U* probabilities were identical, weights for matched and non-matched pairs equalled zero, meaning no distinction was made between matched and non-matched pairs – and thus that the variable was not considered during matching. The impact of this was minimised by inclusion of sufficient other variables in each matching exercise. However, it may have been possible to increase discrimination between linked pairs.

The second set of analyses used estimates obtained with data from a previous matching exercise [15], and better utilised the matching variables. The weakness here was that probability estimates were based on 2006 data, [15] which may not have been appropriate for the earlier time period. However, it was fortunate that there *were* estimates, and only through linkage can the veracity of probability estimates be confirmed.

#### Estimation-maximisation algorithm

The EPICure data set for each epoch was examined in relation to a single day’s worth of HES data at a time. This resulted in different starting points *each day* for the estimation-maximisation algorithm, potentially causing errors to be introduced. It is unknown what effect this differential misclassification may have had, but it is likely this produced an underestimate (i.e. a nullification of effect), as several dates were noted when no convergence of the algorithm was achieved – indicating weights could not be calculated and hence resulting in no matches.

#### EpiLink (Contiero) approach

There was potential for a similar error in the EpiLink analyses [11] because the Contiero algorithm bases estimates of weights on the frequency of responses and estimated error rates [11,14]. This was avoided by specifying these factors in advance.

## Interpretation

Although it was not possible to investigate changes in socioeconomic factors or ethnicity over time using the HES data, it is interesting to note similar increases of around 40 % of all births and of live births in those reported in HES to the 44 % rise in admissions seen in EPICure. This provides some confidence that the EPICure findings are true.

Previous studies have focused on linkage for the entire gestational age range, thus errors at extremes are dissipated. This study demonstrated important data quality concerns in a specific sub-population.

## Generalisability

The findings in relation to the primary objective – to confirm whether there was an increase in births between 1995 and 2006 – are important as they suggest that extremely premature birth is becoming more frequent and build on the observations of the EPICure study [8].

## Conclusions

In conclusion, this study found that HES data are a poor source for information about those born extremely prematurely, with no improvements in data quality seen between 1995 and 2006. However, increases in the absolute numbers of babies born at extremely premature gestations were seen that were in the same direction and of a similar size to those seen in the EPICure studies.

## Ethics approval and consent to participate

The EPICure 2 study was approved by the City and East London REC (*05/Q0605/107*), with additional approval obtained from the Patient Information Advisory Group (*PIAG 3-07(f)/2005*). Permission for this study was granted by the Ethics and Confidentiality Committee of the National Information Governance Board (NIBG) for Health and Social Care (*ECC 1-02(FT3)/2012*). Due to delays in obtaining data and during the analysis, a 6-month extension was granted in March 2013 to permit study resolution.

## Consent for publication

Not applicable.

## Availability of data and materials

The EPICure studies are subject to a data sharing policy that may be downloaded from http://www.epicure.ac.uk. Hospital Episode Statistics are all rights reserved, copyright 2012, and re-used with the permission of The Health and Social Care Information Centre. Statistical code is available from the corresponding author.

## Additional files

Additional file 1 Hospital Episode Statistics. (PDF 56 kb)

Additional file 2 Potential matching of the HES and EPICure data sets. (PDF 94 kb)

Additional file 3 Linkage analysis. (PDF 82 kb)

Additional file 4 Supplementary tables. (PDF 83 kb)

Additional file 5 Supplementary figures. (PDF 86 kb)
