# Supplementary material for: Investigating increased admissions to neonatal intensive care in England between 1995 and 2006: data linkage study using Hospital Episode Statistics
Source: BMC Med Res Methodol. 2016 May 20;16:57. doi: 10.1186/s12874-016-0152-0 (PMC4875750; doi:10.1186/s12874-016-0152-0)
Supplement: Additional file 1 — Hospital Episode Statistics. (PDF 56 kb) [file 12874_2016_152_MOESM1_ESM.pdf]

RESEARCH

Additional file 1: Hospital Episode Statistics

Andrei S Morgan[1*], Neil Marlow[1], Kate Costeloe[2] and Elizabeth S Draper[3]

*Correspondence:
andrei.morgan@ucl.ac.uk
[1]Institute for Womens' Health, UCL, 74 Huntley Street, London, UK
Full list of author information is available at the end of the article

The variables shown in table 1 were provided by the NHS Health and Social Care Information Centre for use in the Admissions Validation Study.

Table 1: Hospital Episode Statistics (HES) variables available for data linkage

Original HES variable name	Data description[a]	Availability	
		1995	2006
HESYEAR	[Financial] year of HES data collection	✓	✓
study_id	Unique identification number for each row	×	✓
neodur	Age of baby (days) at admission	✓	✓
dob	Date of birth (baby)	✓	✓
dob_cfl	Date of birth check flag	✓	✓
ethnos	Ethnic category	✓	✓
newnhsno	NHS number	×	×
encrypted_hesid	Encrypted HES identification number	×	✓
homeadd	[Maternal] home post code	✓	✓
sex	Sex	✓	✓
admidate	Admission date	✓	✓
ADMI_CFL	Admission date check flag	✓	✓
admimeth	Admission method	✓	✓
admisorc	Admission source	✓	✓
disdate	Discharge date	✓	✓
dis_cfl	Discharge date check flag	✓	✓
disdest	Discharge destination	✓	✓
dismeth	Discharge method	✓	✓
classpat	Patient classification	✓	✓
resha	Strategic Health Authority of residence	✓	✓
soal	Super Output Area - Lower	×	✓
soam	Super Output Area - Middle	×	✓
imd04c	Index of Multiple Deprivation (2004) - Crime Domain	×	✓
imd04ed	Index of Multiple Deprivation (2004) - Education Domain	×	✓
imd04hd	Index of Multiple Deprivation (2004) - Housing and Service Domain	×	✓
imd04hs	Index of Multiple Deprivation (2004) - Health and Disability Domain	×	✓
imd04i	Index of Multiple Deprivation (2004) - Income Domain	×	✓
imd04ia	Index of Multiple Deprivation (2004) - Income affecting Adults Domain	×	✓
imd04ic	Index of Multiple Deprivation (2004) - Income affecting Children Domain	×	✓
imd04le	Index of Multiple Deprivation (2004) - Living Environment Domain	×	✓
imd04rk	Index of Multiple Deprivation (2004) - Overall Rank	×	✓
RURURB_IND	Rural-urban indicator	×	✓
dobbaby_1	Date of Birth (baby tail 1)	✓	✓
dobbaby_2	Date of Birth (baby tail 2)	×	×
dobbaby_3	Date of Birth (baby tail 3)	×	×
dobbaby_4	Date of Birth (baby tail 4)	×	×
dobbaby_5	Date of Birth (baby tail 5)	×	×
dobbaby_6	Date of Birth (baby tail 6)	×	×
dobbaby_7	Date of Birth (baby tail 7)	×	×
dobbaby_8	Date of Birth (baby tail 8)	×	×
dobbaby_9	Date of Birth (baby tail 9)	×	×
birordr_1	Birth order (baby tail 1)	✓	✓
birordr_2	Birth order (baby tail 2)	×	×
birordr_3	Birth order (baby tail 3)	×	×
birordr_4	Birth order (baby tail 4)	×	×
birordr_5	Birth order (baby tail 5)	×	×
birordr_6	Birth order (baby tail 6)	×	×

Continued on next page...

Table 1: (Continued.) Variables from Hospital Episode Statistics (HES) available for data linkage with the EPICure cohorts of 1995 and 2006.

**Original HES variable name**	**Data description**[a]	**Availability**	
		**1995**	**2006**
birordr_7	Birth order (baby tail 7)	×	×
birordr_8	Birth order (baby tail 8)	×	×
birordr_9	Birth order (baby tail 9)	×	×
birweit_1	Birth weight (baby tail 1)	✓	✓
birweit_2	Birth weight (baby tail 2)	×	×
birweit_3	Birth weight (baby tail 3)	×	×
birweit_4	Birth weight (baby tail 4)	×	×
birweit_5	Birth weight (baby tail 5)	×	×
birweit_6	Birth weight (baby tail 6)	×	×
birweit_7	Birth weight (baby tail 7)	×	×
birweit_8	Birth weight (baby tail 8)	×	×
birweit_9	Birth weight (baby tail 9)	×	×
delmeth_1	Delivery method (baby tail 1)	✓	✓
delmeth_2	Delivery method (baby tail 2)	×	×
delmeth_3	Delivery method (baby tail 3)	×	×
delmeth_4	Delivery method (baby tail 4)	×	×
delmeth_5	Delivery method (baby tail 5)	×	×
delmeth_6	Delivery method (baby tail 6)	×	×
delmeth_7	Delivery method (baby tail 7)	×	×
delmeth_8	Delivery method (baby tail 8)	×	×
delmeth_9	Delivery method (baby tail 9)	×	×
delplac_1	Delivery place (baby tail 1)	✓	✓
delplac_2	Delivery place (baby tail 2)	×	×
delplac_3	Delivery place (baby tail 3)	×	×
delplac_4	Delivery place (baby tail 4)	×	×
delplac_5	Delivery place (baby tail 5)	×	×
delplac_6	Delivery place (baby tail 6)	×	×
delplac_7	Delivery place (baby tail 7)	×	×
delplac_8	Delivery place (baby tail 8)	×	×
delplac_9	Delivery place (baby tail 9)	×	×
anasdate	First antenatal assessment date	✓	✓
anagest	First antenatal assessment gestation	✓	✓
gestat_1	Gestational age at delivery (baby tail 1)	✓	✓
gestat_2	Gestational age at delivery (baby tail 2)	×	×
gestat_3	Gestational age at delivery (baby tail 3)	×	×
gestat_4	Gestational age at delivery (baby tail 4)	×	×
gestat_5	Gestational age at delivery (baby tail 5)	×	×
gestat_6	Gestational age at delivery (baby tail 6)	×	×
gestat_7	Gestational age at delivery (baby tail 7)	×	×
gestat_8	Gestational age at delivery (baby tail 8)	×	×
gestat_9	Gestational age at delivery (baby tail 9)	×	×
birstat_1	Birth status (baby tail 1)	✓	✓
birstat_2	Birth status (baby tail 2)	×	×
birstat_3	Birth status (baby tail 3)	×	×
birstat_4	Birth status (baby tail 4)	×	×
birstat_5	Birth status (baby tail 5)	×	×
birstat_6	Birth status (baby tail 6)	×	×
birstat_7	Birth status (baby tail 7)	×	×
birstat_8	Birth status (baby tail 8)	×	×
birstat_9	Birth status (baby tail 9)	×	×
matage	Maternal age at delivery	✓	✓
motdob	Mother's date of birth	✓	✓
numbaby	Number of babies delivered (live and still born)	✓	✓
numpreg	Number of previous pregnancies	✓	✓
postdur	Postnatal stay duration	✓	✓
biresus_1	Resuscitation method (baby tail 1)	✓	✓
biresus_2	Resuscitation method (baby tail 2)	×	×
biresus_3	Resuscitation method (baby tail 3)	×	×
biresus_4	Resuscitation method (baby tail 4)	×	×
biresus_5	Resuscitation method (baby tail 5)	×	×
biresus_6	Resuscitation method (baby tail 6)	×	×
biresus_7	Resuscitation method (baby tail 7)	×	×
biresus_8	Resuscitation method (baby tail 8)	×	×
biresus_9	Resuscitation method (baby tail 9)	×	×
sexbaby_1	Sex of baby (baby tail 1)	✓	✓
sexbaby_2	Sex of baby (baby tail 2)	×	×
sexbaby_3	Sex of baby (baby tail 3)	×	×
sexbaby_4	Sex of baby (baby tail 4)	×	×
sexbaby_5	Sex of baby (baby tail 5)	×	×
sexbaby_6	Sex of baby (baby tail 6)	×	×
sexbaby_7	Sex of baby (baby tail 7)	×	×
sexbaby_8	Sex of baby (baby tail 8)	×	×
sexbaby_9	Sex of baby (baby tail 9)	×	×

Continued on next page. . .

Table 1: (Continued.) Variables from Hospital Episode Statistics (HES) available for data linkage with the EPICure cohorts of 1995 and 2006.

Original HES variable name	Data description[a]	Availability 1995	Availability 2006
neocare	Level of neonatal care provided	×	✓
well_baby_ind	Well baby indicator flag	✓	✓
epikey	Record identifier	✓	✓

Variables from Hospital Episode Statistics (HES) available for data linkage with the EPICure cohorts of 1995 and 2006.

Notes:
[a] Taken from the HES data dictionary [1] unless otherwise indicated.

Author details
[1]Institute for Womens' Health, UCL, 74 Huntley Street, London, UK. [2] Homerton Hospital, London, UK. [3] University of Leicester, Leicester, UK.

References
1. The NHS Information Centre for health and social care: Hospital Episode Statistics: Inpatient Data Dictionary. Health & Social Care Information Centre, ??? (2010)
