# Supplementary material for: Investigating increased admissions to neonatal intensive care in England between 1995 and 2006: data linkage study using Hospital Episode Statistics
Source: BMC Med Res Methodol. 2016 May 20;16:57. doi: 10.1186/s12874-016-0152-0 (PMC4875750; doi:10.1186/s12874-016-0152-0)
Supplement: Additional file 2 — Potential matching of the HES and EPICure data sets. (PDF 94 kb) [file 12874_2016_152_MOESM2_ESM.pdf]

RESEARCH

Additional file 2: Potential matching of the HES and EPICure data sets

Andrei S Morgan[1*], Neil Marlow[1], Kate Costeloe[2] and Elizabeth S Draper[3]

*Correspondence: andrei.morgan@ucl.ac.uk
[1]Institute for Womens' Health, UCL, 74 Huntley Street, London, UK
Full list of author information is available at the end of the article

Two broad methods are available for linking data: deterministic, using known uniquely identifying variables (for example, NHS number), or probabilistic, which depends upon statistical likelihood of two rows of data (one from each data set) matching. Deterministic linkage requires an exact match for each variable included in the matching process, hence may be of limited use if there are any ambiguities or errors in the data. Conversely, probabilistic linkage, also known as "fuzzy matching", allows for variation in the matching data, assigning a weight to each comparison pair and then selecting those pairs that rank highest as true matches. Both "match" and "non-match" thresholds may be applied, with optional manual inspection of the data. This method allows for ambiguity in the matching variables and is useful for the present circumstances as the HES data – specifically, for 2006 – are known to contain inaccuracies. [1] For this study, deterministic linkage between the HES and EPICure data sets was carried out in advance by the NHS Health and Social Care Information Centre; however, this was unsuccessful. This project focussed on the probabilistic matching of EPICure and HES data.

Within the total number of births in each time period, the EPICure and HES data may overlap with each other in a number of ways. Figure 1 shows an example of this, using a large blue square to represent all babies born during the study period, which in this case is 2006. The overlapping, slightly smaller box represents all babies who were included in the HES data set: this is likely to be a subset of 'all babies' due to the incomplete coverage already described. The third box, outlined in green, represents those babies who are already included in the EPICure cohorts; and the final, red-edged box represents those babies in HES who meet the EPICure inclusion criteria.

As demonstrated, this leaves five groups of patients who potentially meet the EPICure criteria:

A) Those matched in the EPICure dataset and in HES who already meet the inclusion criteria for EPICure.
B) Those identified by EPICure but with an incorrect gestational age recorded in HES
C) Those not previously known to EPICure but who are identified in HES as meeting the gestational age inclusion criteria.
D) Those in EPICure who have no data at all recorded in HES.
E) Those not identified in either HES or EPICure as having a gestational age of <27 weeks.

Figure 1: **Potential overlap between EPICure 2 and Hospital Episode Statistics (HES) data (2006)**

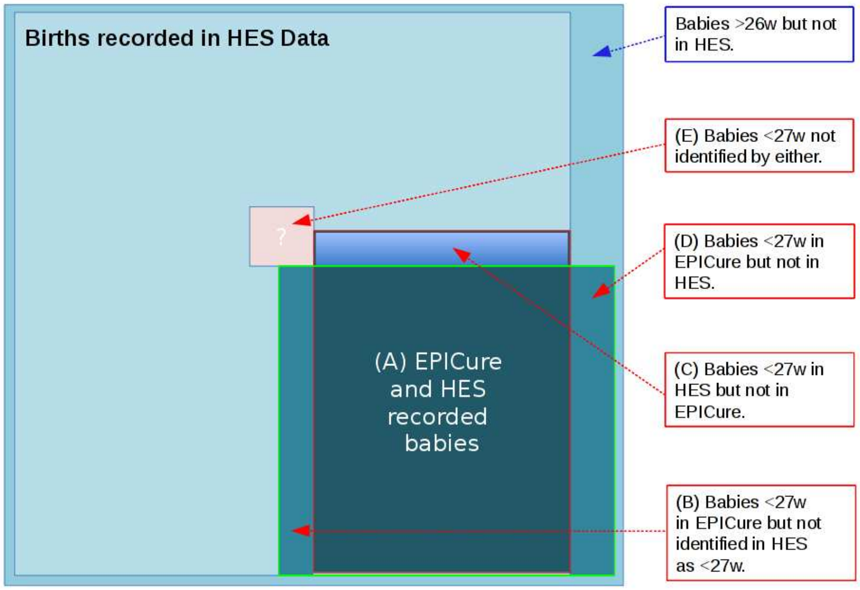

Potential overlap among all births of EPICure 2 and Hospital Episode Statistics (HES) data collection in 2006. The largest blue square represents all babies born during the year. The overlapping, slightly smaller box represents all babies who were included in HES. The green-edged box represents those babies who were included in the EPICure 2 cohort; the red-edged box represents those babies in HES who meet the EPICure 2 inclusion criteria, i.e. have a gestational age of 26^{+6} weeks or less.

Of these, groups A and B (as gestational age and other criteria have already been checked for the EPICure cohorts whereas the HES data are unverified) only need supplementary data from HES. There will be no extra data available for group D. The most important group will be group C: these are subjects (extra to those in the EPICure cohorts) who are identified by HES as being of less than 27 complete weeks gestational age at birth. Group E will be subjects identified from the HES data – and missed from the EPICure data – who are potentially of the correct gestational age, but the gestational age identifying data in HES is either missing or thought to be wrong.

Author details
[1]Institute for Womens' Health, UCL, 74 Huntley Street, London, UK. [2] Homerton Hospital, London, UK. [3] University of Leicester, Leicester, UK.

References
1. Dattani, N., Datta-Nemdharry, P., Macfarlane, A.: Linking maternity data for England, 2005-06: methods and data quality. Health Statistics Quarterly / Office for National Statistics **49**(1), 53–79 (2011). doi:10.1057/hsq.2011.3
