# Supplementary material for: Investigating increased admissions to neonatal intensive care in England between 1995 and 2006: data linkage study using Hospital Episode Statistics
Source: BMC Med Res Methodol. 2016 May 20;16:57. doi: 10.1186/s12874-016-0152-0 (PMC4875750; doi:10.1186/s12874-016-0152-0)
Supplement: Additional file 3 — Linkage analysis. (PDF 82 kb) [file 12874_2016_152_MOESM3_ESM.pdf]

RESEARCH

Additional file 3: Linkage analysis

Andrei S Morgan[1*], Neil Marlow[1], Kate Costeloe[2] and Elizabeth S Draper[3]

*Correspondence:
andrei.morgan@ucl.ac.uk
[1]Institute for Womens' Health, UCL, 74 Huntley Street, London, UK
Full list of author information is available at the end of the article

Blocking variables

As HES data from different years are known to contain errors [1, 2], and because the probability of a single row matching was extremely low (0.1161% for 1995 and 0.4355% in 2006), it was felt that the use of blocking variables would reduce the chances of true matches being identified too much, hence they were not used.

Due to limitations of available computing power coupled with the projected large number of comparison pairs data sets (384,440,012 in 1995 and 1,736,352,750 in 2006), it was not possible to match the EPICure and entire HES data sets at the same time. Instead, for each epoch, the entire EPICure data were sequentially matched with a single day's worth of HES data at a time. For both years, subjects without a date of birth recorded in HES were recoded as being born on "day 0" and then included in the linkage.

Data were analysed using R;[3] bespoke functions were written using the "RecordLinkage" [4] and "ff" [5] packages. Furthermore, the required output was specified in advance: either the list of calculated weights for the entire set of matches *or* the ID numbers for linked pairs above a predefined threshold were produced. This restricted the size of the data actually being handled by the processor at any one time to manageable proportions, while writing anonymised results to disc for future analysis. For each analysis, the function was therefore run twice: first, to obtain the range of weights and numbers of individual values with which to estimate the thresholds for possible links, and then a second time to obtain the corresponding IDs in each data set for weights above the set threshold. Only unique IDs for matches above a pre-defined threshold were retained.

Fellegi & Sunter analysis

Matching was performed for both study epochs in the same way. Each of the three matching algorithms available in the "RecordLinkage" package were used.[4] The most straight forward of these calculates weights (w) stochasticly, based on Fellegi and Sunter's work, whereby both the M probability (i.e. that both records of a pair are from the same subject) and U probabilities (where records in a pair belong to different subjects) are specified in advance. [4] The calculations are performed as follows:

$$w = \left\{ \begin{array}{ll} \log_2(M/U) & \text{if records are the same;} \\ \log_2(1-m)/(1-u) & \text{if records are different.} \end{array} \right\} \qquad (1)$$

Values chosen for M and U probabilities may have an important impact on the results thus should be chosen carefully. Dattani et al [1] provide some data on which

the 2006 estimates of these values may be based. However, as not all of the variables to be used for matching had prior estimates, it was decided to perform one round of matching using best-guess values, and a second round of matching using the Dattani et al estimates. The best guess values were derived using the following rules:

***M*-probability** based on the estimated accuracy of record completion.

***U*-probability** based on chance agreement: the likelihood that two subjects would match if the subjects were chosen randomly.

For the M-probabiliities, date of birth, mother's age at delivery, baby sex and number of babies were considered to have a high probability ($\geq$ 90%) of having been entered correctly; for other variables, the estimated probabilities varied as low as 20%. Best guess U-probabilities for date of birth and death were set at $1/365 = 0.00274$, and for discharge date, 1/500, as HES is likely to be discrepant from EPICure data in this respect; for birth order, number of babies and number of previous pregnancies at 90% as pregnancies of lower birth are more common, as are lower parity women; and sex at 0.49 so as to account for those of indeterminate sex. Gestational age at birth and maternal age were based on approximate number of categories with a slight adjustment for unequal distributions. Birth weight was assigned a U-probability of 1/1000, i.e. 0.001. The full set of values, along with corresponding weights, are shown in table 1.

Table 1: Probability estimates for linkage analyses

**Matching variable**	**Baseline best guesses**				**Dattani et al [1] estimate**			
	m	u	w_{m} [a]	w_{nm} [b]	m	u	w_{m} [a]	w_{nm} [b]
Date of birth	0.90	0.00274	5.794	-2.3	0.7405	0.0015	6.202	-1.347
GA at birth	0.80	0.02	3.689	-1.589	0.4941	0.0494	2.3028	-0.6308
Sex	0.999	0.49	0.7123	-6.2344	0.7208	0.0062	4.756	-1.270
Discharge date	0.20	0.002	4.6052	-0.2211	—	—	—	—
Date of death [a]	0.20	0.00274	4.2904	-0.2204	0.30	0.002	5.0106	-0.3547
Birth weight	0.60	0.001	6.3969	-0.9153	0.7405	0.0074	4.606	-1.342
Birth order	0.87	0.95	-0.08797	0.95551	0.8153	0.0033	5.510	-1.686
Delivery method [a]	0.80	0.80	0	0	0.67	0.1	1.902	-1.003
Ethnic category	0.20	0.10	0.6931	-0.1178	0.7308	0.095	2.040	-1.212
Mother's age at delivery	0.95	0.05	2.944	-2.944	—	—	—	—
Mother's date of birth	0.90	0.0001	9.105	-2.302	—	—	—	—
Postcode	0.90	0.001	6.802	-2.302	0.9291	0.065	2.660	-2.579
Number of previous pregnancies	0.60	0.90	-0.4055	1.3863	—	—	—	—
Number of babies	0.95	0.95	0	0	0.8153	0.0033	5.510	-1.686

[a] w_{m} = weight if pairs match.

[b] w_{nm} = weight if pairs do not match.

[c] Date of death and delivery method were both modified using an adjusted best guess for the second linkage analysis performed using estimates from Dattani et al.

Probability estimates for linkage analyses between Hospital Episode Statistics and EPICure data based on best guesses and prior knowledge (adapted from data linkage performed by Dattani et al between Hospital Episode Statistics (HES) and NHS Numbers 4 Babies data sets).[1]

In the comparison round of matching, using the Dattani estimates, data were available for date of birth, postcode, number of babies in the pregnancy, sex, birth weight, gestational age and ethnicity; of these, absolute numbers were provided for number of concordant and discordant pairs for number of births per pregnancy and sex, and percentages of concordant pairs for the remaining variables. It was therefore

possible to calculate probabilities for these variables using equations 2 and 3 (C = concordance rate, D = discordance rate, and P_{nm} = percentage not missing):

$$M = CP_{\text{nm}} \tag{2}$$

$$U = CDP_{\text{nm}} \tag{3}$$

Where no prior information was available from the Dattani et al estimates for variables to be used in the matching, the best guess values were used in supplement.

Contiero analysis

The second method of matching uses the algorithm designed by Contiero, on which the *EpiLink* software is based. [4] For this method, the overall weight (w_o) for each subject-pair can be calculated as:

$$w_o = \frac{\sum w_i s_i(x_i^1, x_i^2)}{\sum w_i} \tag{4}$$

where s_i is the value of the comparison between the ith records from each of the data sets x and y, and w_i is the weight attached to that particular (variable) comparison. Weights are assigned in the range $0 \leq w \leq 1$.[4, 6] Both error rates and frequencies used to derive the variable weights were explicitly set according to the default values for the overall data sets.

Estimation-maximisation analysis

The final method of matching uses an automated method to assign weights based on maximum likelihood, and is known as the *estimation-maximisation* algorithm. [4] This did not require any parameters other than the names of the data sets to be passed to it.

Main comparisons and cut-off points

There were $477,898 \times 668 = 325,118,940$ potential comparison pairs in 1995, and $631,401 \times 2,750 = 1,736,352,750$ pairs in 2006. It was not possible (or desirable) to save all this information as the vast majority were false matches. Therefore, each linkage method required a preliminary review of the calculated weights in order to select appropriate cut-offs above which to retain linked or potentially linked data pairs (one each from the HES and EPICure data sets). Cut-off points were selected according to where a "reasonable" number of linked pairs was obtained.

Author details
[1]Institute for Womens' Health, UCL, 74 Huntley Street, London, UK. [2] Homerton Hospital, London, UK. [3] University of Leicester, Leicester, UK.

References

1. Dattani, N., Datta-Nemdharry, P., Macfarlane, A.: Linking maternity data for England, 2005-06: methods and data quality. Health Statistics Quarterly / Office for National Statistics **49**(1), 53–79 (2011). doi:10.1057/hsq.2011.3
2. Dattani, N., Datta-Nemdharry, P., Macfarlane, A.: Linking maternity data for England 2007: methods and data quality. Health Statistics Quarterly / Office for National Statistics **53**(Spring), 4–21 (2012)
3. R Core Team, R Foundation for Statistical Computing: R: A Language and Environment for Statistical Computing. (2013). http://www.R-project.org/
4. Borg, A., Sariyar, M.: Package “RecordLinkage”. (2012). https://r-forge.r-project.org/projects/recordlinkage/
5. Adler, D., Gläser, C., Nenadic, O., Oehlschlägel, J., Zucchini, W.: Package 'ff'. (2013). http://cran.r-project.org/web/packages/ff/index.html
6. Contiero, P., Tittarelli, A., Tagliabue, G., Maghini, A., Fabiano, S., Crosignani, P., Tessandori, R.: The EpiLink record linkage software: presentation and results of linkage test on cancer registry files. Methods of Information in Medicine **44**(1), 66–71 (2005). doi:10.1267/METH05010066
