# Supplementary material for: Investigating increased admissions to neonatal intensive care in England between 1995 and 2006: data linkage study using Hospital Episode Statistics
Source: BMC Med Res Methodol. 2016 May 20;16:57. doi: 10.1186/s12874-016-0152-0 (PMC4875750; doi:10.1186/s12874-016-0152-0)
Supplement: Additional file 4 — Supplementary tables. (PDF 83 kb) [file 12874_2016_152_MOESM4_ESM.pdf]

RESEARCH

Additional file 4: Supplementary tables

Andrei S Morgan[1*], Neil Marlow[1], Kate Costeloe[2] and Elizabeth S Draper[3]

*Correspondence:
andrei.morgan@ucl.ac.uk
[1]Institute for Womens' Health, UCL, 74 Huntley Street, London, UK
Full list of author information is available at the end of the article

Table S1: Spread of values amongst matching variables

Variable	Level	HES 1995	EPICure	HES 2006	EPICure 2
		N (%)	N (%)	N (%)	N (%)
Month of birth					
	Jan	45804 (7.96)	—	50192 (7.95)	231 (8.40)
	Feb	43000 (7.47)	—	46817 (7.41)	209 (7.60)
	Mar	47591 (8.27)	56 (8.38)	51436 (8.15)	234 (8.51)
	Apr	46631 (8.10)	66 (9.88)	50261 (7.96)	240 (8.73)
	May	49634 (8.62)	66 (9.88)	54118 (8.57)	218 (7.93)
	Jun	49524 (8.61)	76 (11.38)	53277 (8.44)	264 (9.60)
	Jul	50048 (8.70)	62 (9.28)	54016 (8.55)	225 (8.18)
	Aug	49236 (8.56)	65 (9.73)	55377 (8.77)	210 (7.64)
	Sep	49224 (8.55)	55 (8.23)	55725 (8.83)	263 (9.56)
	Oct	48975 (8.51)	69 (10.33)	56435 (8.94)	238 (8.65)
	Nov	45475 (7.90)	66 (9.88)	52284 (8.28)	205 (7.45)
	Dec	41560 (7.22)	87 (13.02)	47198 (7.48)	213 (7.75)
	Missing	8807 (1.53)	0 (0.00)	4265 (0.68)	0 (0.00)
GA at birth (weeks)					
	<20	0 (0.00)	—	95 (0.02)	24 (0.87)
	20	28 (0.00)	—	36 (0.01)	34 (1.24)
	21	44 (0.01)	2 (0.30)	66 (0.01)	66 (2.40)
	22	82 (0.01)	16 (2.40)	87 (0.01)	151 (5.49)
	23	156 (0.03)	115 (17.22)	212 (0.03)	322 (11.71)
	24	369 (0.06)	244 (36.53)	384 (0.06)	637 (23.16)
	25	398 (0.07)	291 (43.56)	682 (0.11)	686 (24.95)
	26-29	2519 (0.44)	—	6446 (1.02)	820 (29.82)
	30-34	10727 (1.86)	—	7813 (1.24)	3 (0.11)
	35-39	173806 (30.20)	—	129294 (20.48)	0 (0.00)
	40+	223374 (38.81)	—	150170 (23.78)	0 (0.00)
	Missing	164006 (28.50)	0 (0.00)	336116 (53.23)	7 (0.25)
Sex					
	Female	278267 (48.35)	359 (53.74)	307080 (48.63)	1422 (51.71)
	Male	292844 (50.88)	309 (46.26)	321119 (50.86)	1319 (47.96)
	Indeterminate	1782 (0.31)	—	—	—
	Missing	2616 (0.45)	0 (0.00)	3202 (0.51)	9 (0.33)
Birth weight (g)					
	<500	2634 (0.46)	27 (4.04)	520 (0.08)	455 (16.55)
	500-999	1896 (0.33)	638 (95.51)	1904 (0.30)	2117 (76.98)
	1000-1499	3455 (0.60)	3 (0.45)	2833 (0.45)	149 (5.42)
	1500-1999	6263 (1.09)	0 (0.00)	5913 (0.94)	3 (0.11)
	2000-2499	19528 (3.39)	0 (0.00)	17477 (2.77)	0 (0.00)
	2500-2999	72098 (12.53)	—	59906 (9.49)	—
	3000-3499	152613 (26.52)	—	121699 (19.27)	—
	3500-3999	119605 (20.78)	—	96202 (15.24)	—
	4000-4499	38222 (6.64)	—	31414 (4.98)	—
	4500-4999	5860 (1.02)	—	4944 (0.78)	—
	5000+	694 (0.12)	—	622 (0.10)	—
	Missing	152641 (26.52)	0 (0.00)	287967 (45.61)	26 (0.95)
Birth order					
	1	318895 (55.41)	562 (84.13)	401473 (63.58)	2428 (88.29)
	>1	5896 (1.02)	106 (15.87)	5338 (0.85)	322 (11.71)
	Missing	250718 (43.56)	0 (0.00)	224590 (35.57)	0 (0.00)
Maternal age (years)					
	<16	940 (0.16)	3 (0.45)	682 (0.11)	12 (0.44)
	16-19	21343 (3.71)	49 (7.34)	23494 (3.72)	219 (7.96)
	20-29	190184 (33.05)	326 (48.80)	162499 (25.74)	1260 (45.82)
	30-39	138764 (24.11)	276 (41.32)	158740 (25.14)	1116 (40.58)
	40-49	9255 (1.61)	10 (1.50)	12583 (1.99)	130 (4.73)
	50+	24 (0.00)	0 (0.00)	35 (0.01)	5 (0.18)
	Missing	214999 (37.36)	4 (0.60)	273368 (43.30)	8 (0.29)

Spread of values amongst matching variables used in the linkage exercises performed between EPICure and Hospital Episode Statistics (HES) data for 1995 and 2006. (**EPICure** $n = 668$ (March – December); **EPICure 2** $n = 2,750$; **HES (1995)** $= 575,509$ for the entire year; **HES (2006)** $n = 631,401$.)

Table S2: Birth weight v gestational age in HES 2006 data

**Birth weight category**	**Gestational age (weeks)**													
	< 20	**20**	**21**	**22**	**23**	**24**	**25**	**26**	**27-29**	**30-34**	**35-39**	**40+**	**Missing**	**Total**
<500	16 (0.003)	17 (0.003)	28 (0.004)	28 (0.004)	21 (0.003)	35 (0.006)	25 (0.004)	15 (0.002)	28 (0.004)	14 (0.002)	64 (0.010)	86 (0.014)	143 (0.023)	520 (0.082)
500-999	10 (0.002)	1 (0.000)	3 (0.000)	19 (0.003)	101 (0.016)	218 (0.035)	226 (0.036)	213 (0.034)	345 (0.055)	86 (0.014)	11 (0.002)	9 (0.001)	662 (0.105)	1904 (0.302)
1000-1499	13 (0.002)	11 (0.002)	14 (0.002)	4 (0.001)	5 (0.001)	6 (0.001)	14 (0.002)	41 (0.006)	685 (0.108)	934 (0.148)	92 (0.015)	23 (0.004)	991 (0.157)	2833 (0.449)
1500-1999	0 (0.000)	0 (0.000)	8 (0.001)	17 (0.003)	22 (0.003)	13 (0.002)	31 (0.005)	4 (0.001)	86 (0.014)	2355 (0.373)	1231 (0.195)	70 (0.011)	2076 (0.329)	5913 (0.936)
2000-2499	1 (0.000)	0 (0.000)	1 (0.000)	4 (0.001)	15 (0.002)	37 (0.006)	89 (0.014)	64 (0.010)	82 (0.013)	2038 (0.323)	8289 (1.313)	1015 (0.161)	5842 (0.925)	17477 (2.768)
2500-2999	1 (0.000)	2 (0.000)	1 (0.000)	0 (0.000)	6 (0.001)	13 (0.002)	137 (0.022)	216 (0.034)	667 (0.106)	568 (0.090)	27750 (4.395)	11464 (1.816)	19081 (3.022)	59906 (9.488)
3000-3499	3 (0.000)	2 (0.000)	2 (0.000)	0 (0.000)	3 (0.000)	6 (0.001)	78 (0.012)	222 (0.035)	1580 (0.250)	152 (0.024)	38932 (6.166)	41831 (6.625)	38888 (6.159)	121699 (19.274)
3500-3999	1 (0.000)	0 (0.000)	0 (0.000)	0 (0.000)	0 (0.000)	1 (0.000)	14 (0.002)	87 (0.014)	1261 (0.200)	61 (0.010)	20293 (3.214)	43579 (6.902)	30905 (4.895)	96202 (15.236)
4000-4499	4 (0.001)	0 (0.000)	0 (0.000)	1 (0.000)	0 (0.000)	1 (0.000)	7 (0.001)	23 (0.004)	405 (0.064)	10 (0.002)	4516 (0.715)	16275 (2.578)	10172 (1.611)	31414 (4.975)
4500-4999	2 (0.000)	0 (0.000)	0 (0.000)	0 (0.000)	0 (0.000)	0 (0.000)	1 (0.000)	0 (0.000)	60 (0.010)	1 (0.000)	630 (0.100)	2684 (0.425)	1566 (0.248)	4944 (0.783)
5000+	12 (0.002)	1 (0.000)	0 (0.000)	0 (0.000)	0 (0.000)	2 (0.000)	0 (0.000)	1 (0.000)	3 (0.000)	4 (0.001)	96 (0.015)	292 (0.046)	211 (0.033)	622 (0.099)
Missing	32 (0.005)	2 (0.000)	9 (0.001)	14 (0.002)	39 (0.006)	52 (0.008)	60 (0.010)	87 (0.014)	271 (0.043)	1590 (0.252)	27390 (4.338)	32842 (5.201)	225579 (35.727)	287967 (45.608)
Total	95 (0.015)	36 (0.006)	66 (0.010)	87 (0.014)	212 (0.034)	384 (0.061)	682 (0.108)	973 (0.154)	5473 (0.867)	7813 (1.237)	129294 (20.477)	150170 (23.784)	336116 (53.233)	(100.000)

Numbers of subjects (percentage of overall data set) according to birth weight (g) by gestational age (weeks), as recorded in the 2006 Hospital Episode Statistics data set.

Table S3: Number of pairs matched using guestimate probabilities (2006)

Cut off weight	N pairs	N EPICure	% EPICure	N HES	% HES
10	44719	2729	99.24	36025	5.71
11	40363	2701	98.22	34741	5.50
12	2459	1569	57.05	1811	0.29
13	2445	1567	56.98	1806	0.29
16	2130	1455	52.91	1608	0.25
17	2082	1435	52.18	1576	0.25
19	2072	1430	52.00	1572	0.25
20	2004	1394	50.69	1526	0.24
21	1969	1385	50.36	1517	0.24
22	1895	1362	49.53	1489	0.24
23	1886	1360	49.45	1488	0.24
24	1515	1128	41.02	1189	0.19
26	1499	1121	40.76	1181	0.19
27	1475	1119	40.69	1179	0.19
28	1474	1118	40.65	1178	0.19
29	1460	1108	40.29	1168	0.18
30	1411	1086	39.49	1149	0.18
31	1168	917	33.35	970	0.15
32	1165	915	33.27	968	0.15
33	1127	889	32.33	934	0.15
34	994	788	28.65	836	0.13
35	972	777	28.25	828	0.13
36	970	775	28.18	826	0.13
37	726	578	21.02	614	0.10
38	724	576	20.95	612	0.10
39	710	565	20.55	601	0.10
41	675	539	19.60	571	0.09
42	559	446	16.22	476	0.08
43	494	430	15.64	461	0.07
44	266	233	8.47	259	0.04
45	265	233	8.47	258	0.04
46	249	218	7.93	242	0.04
53	126	117	4.25	122	0.02
54	102	93	3.38	98	0.02
55	0	0	0.00	0	0.00

Table of the number of pairs matched in 2006 from each data set for differing cutoffs in the value of the weight calculated by the Fellegi-Sunter (guestimate) method of data linkage.

Table S4: Number of pairs matched using Dattani estimates (1995)

Cut off weight	N pairs	N EPICure	% EPICure	N HES	% HES
35	29453	453	67.81	16385	3.37
36	3674	409	61.23	3540	0.73
37	3649	400	59.88	3518	0.72
38	1064	299	44.76	1049	0.22
39	1044	290	43.41	1029	0.21
40	920	277	41.47	915	0.19
41	905	270	40.42	900	0.18
42	313	198	29.64	311	0.06
43	254	169	25.30	252	0.05
44	240	160	23.95	240	0.05
45	205	147	22.01	205	0.04
46	87	85	12.72	87	0.02
47	85	83	12.43	85	0.02
48	80	78	11.68	80	0.02
49	69	69	10.33	69	0.01
50	51	51	7.63	51	0.01
51	49	49	7.34	49	0.01
52	40	40	5.99	40	0.01
53	38	38	5.69	38	0.01
54	33	33	4.94	33	0.01
55	29	29	4.34	29	0.01
56	29	29	4.34	29	0.01
57	27	27	4.04	27	0.01
58	18	18	2.69	18	0
59	13	13	1.95	13	0
61	13	13	1.95	13	0
62	5	5	0.75	5	0
63	3	3	0.45	3	0
65	3	3	0.45	3	0
66	0	0	0	0	0

Table of the number of pairs matched in 1995 from each data set for differing cutoffs in the value of the weight calculated by the Fellegi-Sunter method of data linkage using estimates based on Dattani et al.[1]

Table S5: Number of pairs matched using Dattani estimates (2006)

Cut off weight	N pairs	N EPICure	% EPICure	N HES	% HES
15	53413	2709	98.51	40104	6.35
16	38608	2672	97.16	32232	5.10
17	38020	2662	96.80	32114	5.09
18	37857	2658	96.65	32051	5.08
19	4497	1897	68.98	3129	0.50
20	4397	1885	68.55	3078	0.49
21	3614	1741	63.31	2489	0.39
22	3605	1741	63.31	2489	0.39
23	3582	1736	63.13	2469	0.39
24	2362	1512	54.98	1814	0.29
25	2358	1511	54.95	1812	0.29
26	2279	1488	54.11	1776	0.28
27	2200	1449	52.69	1717	0.27
28	2172	1432	52.07	1701	0.27
29	2159	1431	52.04	1700	0.27
30	1440	1089	39.60	1144	0.18
31	1439	1089	39.60	1144	0.18
32	1427	1082	39.35	1137	0.18
33	1404	1073	39.02	1129	0.18
34	1169	923	33.56	980	0.16
35	1162	920	33.45	974	0.15
36	1118	899	32.69	933	0.15
37	1116	899	32.69	933	0.15
38	930	748	27.20	781	0.12
39	821	661	24.04	692	0.11
40	817	658	23.93	690	0.11
41	772	624	22.69	653	0.10
42	540	457	16.62	488	0.08
43	431	368	13.38	398	0.06
44	431	368	13.38	398	0.06
45	385	338	12.29	362	0.06
46	300	253	9.20	277	0.04
47	300	253	9.20	277	0.04
48	278	231	8.40	255	0.04
49	242	206	7.49	224	0.04
50	242	206	7.49	224	0.04
51	141	114	4.15	127	0.02
52	83	70	2.55	75	0.01
53	38	37	1.35	38	0.01
56	38	37	1.35	38	0.01
57	26	26	0.95	26	0
60	26	26	0.95	26	0
61	2	2	0.07	2	0
63	2	2	0.07	2	0
64	0	0	0	0	0

Table of the number of pairs matched in 2006 from each data set for differing cutoffs in the value of the weight calculated by the Fellegi-Sunter method of data linkage using estimates based on Dattani et al.[1]

Table S6: Number of pairs matched using the EpiLink algorithm (2006)

Cut off weight	N pairs	N EPICure	% EPICure	N HES	% HES
0.35	6323	2162	78.62	5608	0.89
0.40	2746	1542	56.07	2405	0.38
0.45	1573	1225	44.55	1328	0.21
0.50	1413	1135	41.27	1198	0.19
0.55	1180	965	35.09	1018	0.16
0.60	1125	922	33.53	975	0.15
0.65	928	774	28.15	805	0.13
0.70	623	514	18.69	545	0.09
0.75	538	447	16.25	478	0.08
0.80	264	237	8.62	264	0.04
0.85	137	129	4.69	137	0.02
0.90	122	116	4.22	122	0.02
0.95	0	0	0.00	0	0.00

Table of the number of pairs matched in 2006 from each data set for differing cutoffs in the value of the weight calculated by the EpiLink (Contiero) method of data linkage.

Table S7: Number of pairs matched using the estimation-maximisation algorithm (1995)

Cut off weight	N pairs	N EPICure	% EPICure	N HES	% HES
25	644	232	34.73	502	0.10
26	541	212	31.74	421	0.09
27	429	189	28.29	348	0.07
28	294	175	26.20	241	0.05
29	231	155	23.20	201	0.04
30	177	139	20.81	161	0.03
31	153	132	19.76	147	0.03
32	140	123	18.41	135	0.03
33	136	120	17.96	132	0.03
34	134	118	17.66	130	0.03
35	129	114	17.07	125	0.03
36	121	108	16.17	117	0.02
37	114	101	15.12	110	0.02
38	107	96	14.37	103	0.02
39	99	90	13.47	95	0.02
40	90	82	12.28	86	0.02
41	80	77	11.53	79	0.02
42	74	73	10.93	73	0.01
43	66	66	9.88	66	0.01
44	61	61	9.13	61	0.01
45	58	58	8.68	58	0.01
46	53	53	7.93	53	0.01
47	48	48	7.19	48	0.01
48	43	43	6.44	43	0.01
49	37	37	5.54	37	0.01
50	34	34	5.09	34	0.01

Table of the number of pairs matched from each data in 1995 set for differing cutoffs in the value of the weight calculated by the estimation-maximisation method of data linkage.

Table S8: Number of pairs matched using the estimation-maximisation algorithm (2006)

Cut off weight	N pairs	N EPICure	% EPICure	N HES	% HES
10	57519	2712	98.62	37589	5.95
11	45110	2712	98.62	34287	5.43
12	40419	2706	98.40	33051	5.23
13	37885	2686	97.67	32260	5.11
14	35164	2683	97.56	30017	4.75
15	33606	2678	97.38	28588	4.53
16	33570	2677	97.35	28558	4.52
17	33554	2677	97.35	28542	4.52
18	33540	2677	97.35	28529	4.52
19	33391	2677	97.35	28428	4.50
20	31450	2644	96.15	27453	4.35
21	22960	2483	90.29	20527	3.25
22	1981	1362	49.53	1645	0.26
23	1766	1265	46.00	1450	0.23
24	1687	1225	44.55	1395	0.22
25	1473	1113	40.47	1219	0.19
26	1340	1036	37.67	1106	0.18
27	1292	1009	36.69	1071	0.17
28	1247	980	35.64	1036	0.16
29	1201	939	34.15	993	0.16
30	1113	871	31.67	927	0.15
31	1056	835	30.36	883	0.14
32	999	799	29.05	845	0.13
33	953	771	28.04	816	0.13
34	902	729	26.51	776	0.12
35	856	686	24.95	734	0.12
36	806	645	23.45	695	0.11
37	747	599	21.78	647	0.10
38	685	550	20.00	597	0.09
39	638	513	18.65	553	0.09
40	555	449	16.33	483	0.08
41	499	408	14.84	438	0.07
42	455	374	13.60	400	0.06
43	414	339	12.33	359	0.06
44	400	325	11.82	345	0.05
45	388	315	11.45	336	0.05
46	382	310	11.27	330	0.05
47	356	289	10.51	307	0.05
48	335	277	10.07	296	0.05
49	304	259	9.42	279	0.04
50	277	239	8.69	259	0.04
51	232	207	7.53	225	0.04
52	206	184	6.69	202	0.03
53	173	155	5.64	172	0.03
54	137	131	4.76	137	0.02
55	117	113	4.11	117	0.02
56	100	96	3.49	100	0.02
57	89	85	3.09	89	0.01
58	77	73	2.65	77	0.01
59	70	66	2.40	70	0.01
60	65	61	2.22	65	0.01

Continued on next page...

Table S8: Number of pairs at different cut-offs obtained from the estimation-maximisation algorithm in 2006 (Continued).

Cut off weight	N pairs	N EPICure	% EPICure	N HES	% HES
61	61	57	2.07	61	0.01
62	58	54	1.96	58	0.01
63	56	52	1.89	56	0.01
64	48	45	1.64	48	0.01
65	41	38	1.38	41	0.01
66	36	33	1.20	36	0.01
67	27	25	0.91	27	0.00
68	15	14	0.51	15	0.00
69	9	8	0.29	9	0.00
70	2	2	0.07	2	0.00

Table of the number of pairs matched from each data set in 2006 for differing cutoffs in the value of the weight calculated by the estimation-maximisation method of data linkage.

Author details

[1]Institute for Womens' Health, UCL, 74 Huntley Street, London, UK. [2] Homerton Hospital, London, UK. [3] University of Leicester, Leicester, UK.

References

1. Dattani, N., Datta-Nemdharry, P., Macfarlane, A.: Linking maternity data for England, 2005-06: methods and data quality. Health Statistics Quarterly / Office for National Statistics **49**(1), 53–79 (2011). doi:10.1057/hsq.2011.3
