# Supplementary material for: Investigating increased admissions to neonatal intensive care in England between 1995 and 2006: data linkage study using Hospital Episode Statistics
Source: BMC Med Res Methodol. 2016 May 20;16:57. doi: 10.1186/s12874-016-0152-0 (PMC4875750; doi:10.1186/s12874-016-0152-0)
Supplement: Additional file 5 — Supplementary figures. (PDF 86 kb) [file 12874_2016_152_MOESM5_ESM.pdf]

RESEARCH

Additional file 5: Supplementary figures

Andrei S Morgan[1*], Neil Marlow[1], Kate Costeloe[2] and Elizabeth S Draper[3]

*Correspondence:
andrei.morgan@ucl.ac.uk
[1]Institute for Womens' Health, UCL, 74 Huntley Street, London, UK
Full list of author information is available at the end of the article

Author details
[1]Institute for Womens' Health, UCL, 74 Huntley Street, London, UK. [2] Homerton Hospital, London, UK. [3] University of Leicester, Leicester, UK.

References

1. Dattani, N., Datta-Nemdharry, P., Macfarlane, A.: Linking maternity data for England, 2005-06: methods and data quality. Health Statistics Quarterly / Office for National Statistics **49**(1), 53–79 (2011). doi:10.1057/hsq.2011.3
2. Borg, A., Sariyar, M.: Package "RecordLinkage". (2012). https://r-forge.r-project.org/projects/recordlinkage/
3. Contiero, P., Tittarelli, A., Tagliabue, G., Maghini, A., Fabiano, S., Crosignani, P., Tessandori, R.: The EpiLink record linkage software: presentation and results of linkage test on cancer registry files. Methods of Information in Medicine **44**(1), 66–71 (2005). doi:10.1267/METH05010066

Figure S1: **Density distribution of weights – stochastic linkage (Dattani estimates)**

(a) 1995 baseline best guesses

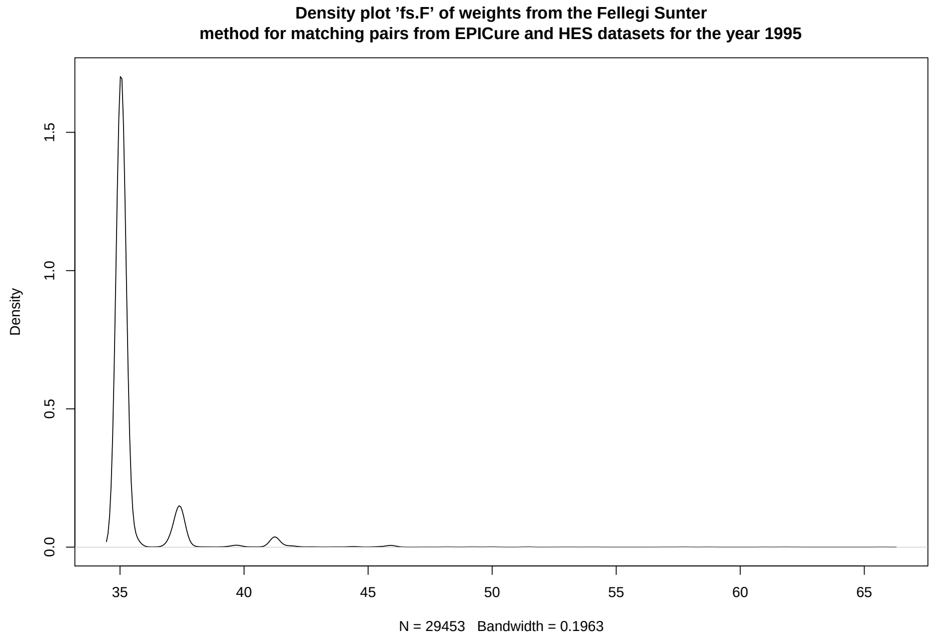

(b) 2006 baseline best guesses

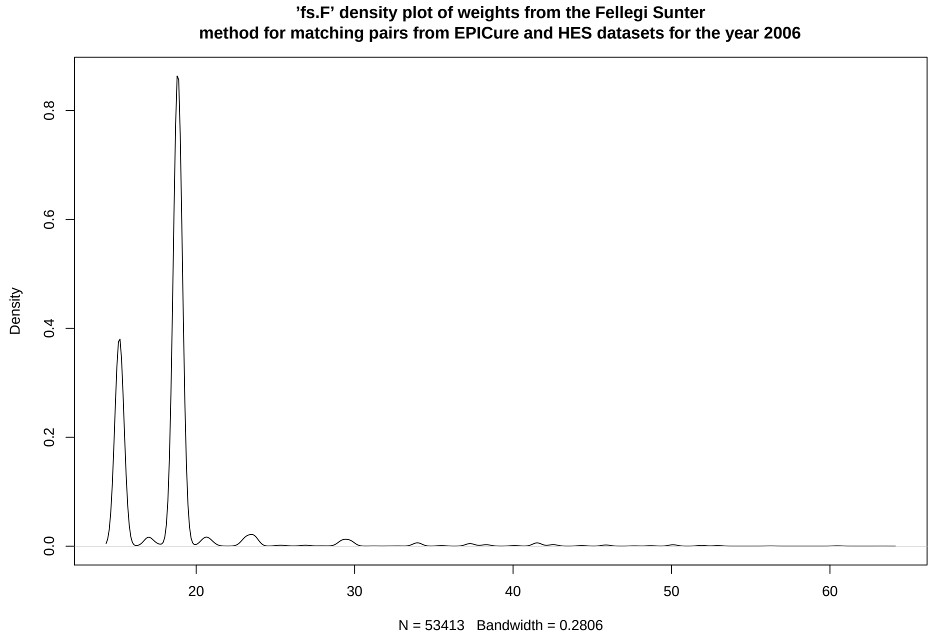

Density distribution of weights from the stochastic linkage analyses using probabilities based on Dattani et al.[1] Axes are not to the same scale.

Figure S2: **Individual matches according to weight – stochastic analysis (Dattani estimates)**

(a) 1995 (b) 2006

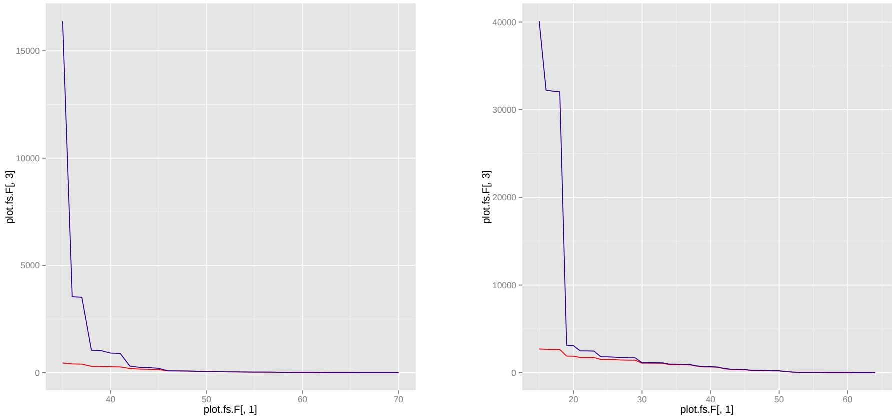

Numbers of individual matches according to weight from each of the Hospital Episode Statistics (HES) (blue line) and EPICure (red line) data sets in the stochastic linkage analysis using probabilities based on Dattani et al.[1] "Weight" is on the x-axis, number of matches on the y-axis; axes are not to the same scale.

Figure S3: **Density distribution of weights – EpiLink algorithm**

(a) 1995

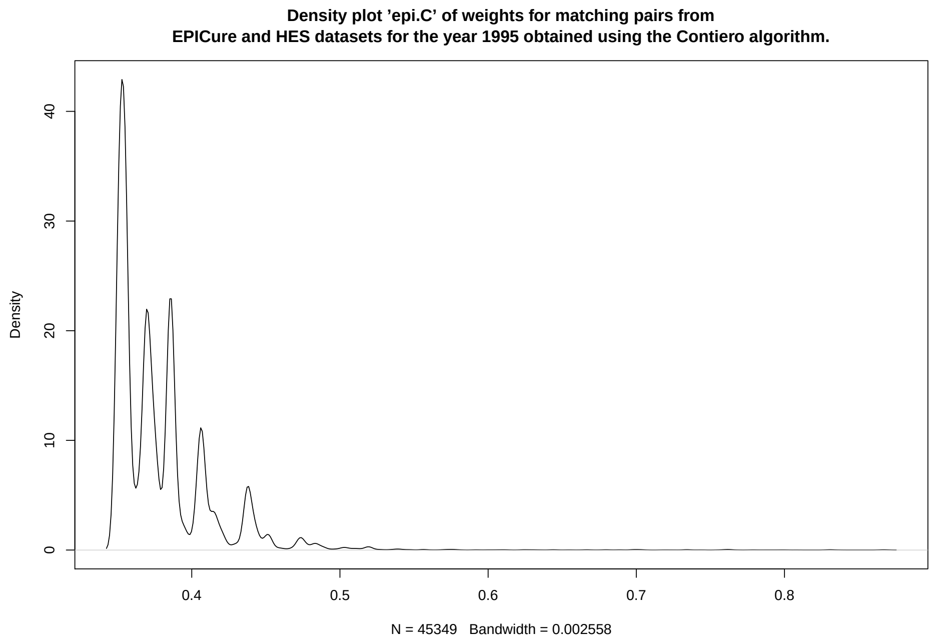

(b) 2006

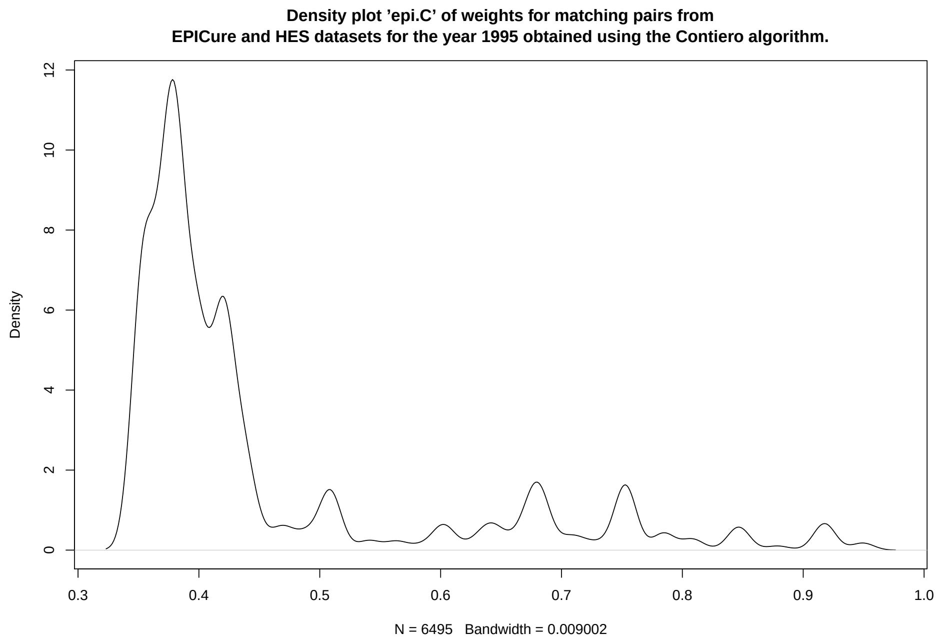

Density distribution of weights from the linkage analyse using Contiero's EpiLink algorithm.[2, 3] Axes are not to the same scale.

Figure S4: **Individual matches according to weight – EpiLink algorithm**

(a) 1995 (b) 2006

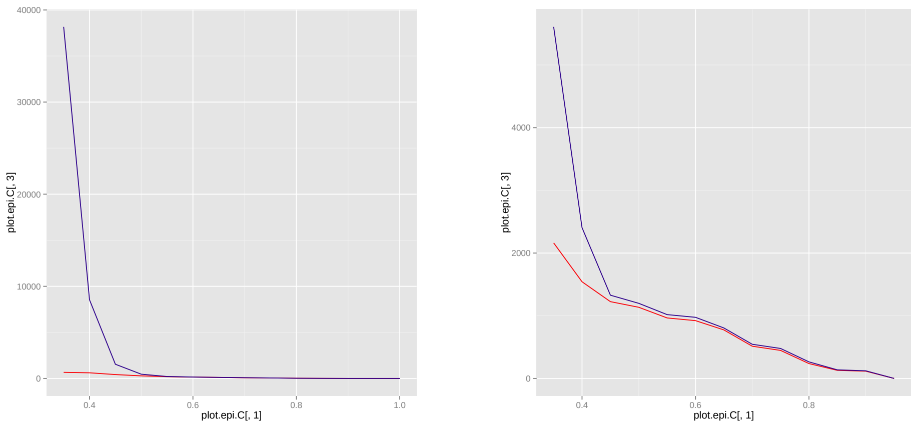

Numbers of individual matches according to weight from each of the Hospital Episode Statistics (HES) (blue line) and EPICure (red line) data sets in the linkage analysis performed using Contiero's EpiLink algorithm.[2, 3] "Weight" is on the x-axis, number of matches on the y-axis; axes are not to the same scale.

Figure S5: **Individual matches according to weight – estimation-maximisation algorithm**

(a) 1995 (b) 2006

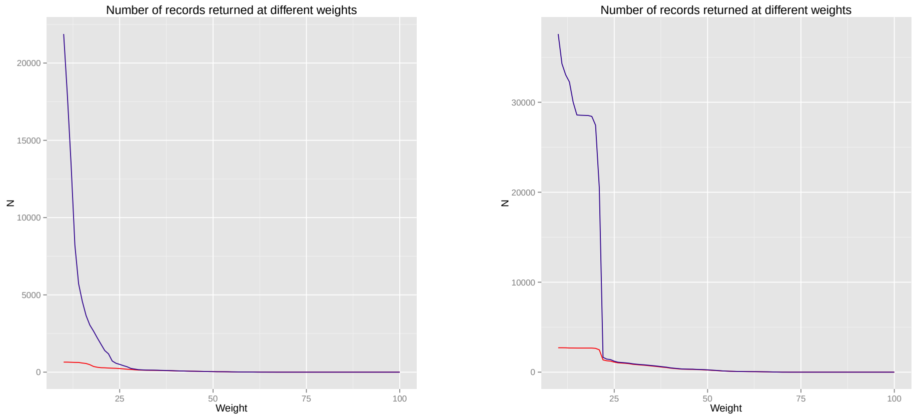

Numbers of individual matches according to weight from each of the Hospital Episode Statistics (HES) (blue line) and EPICure (red line) data sets in the linkage analysis based on the estimation-maximisation algorithm. "Weight" is on the x-axis, number of matches on the y-axis; axes are not all to the same scale.

Figure S6: **Density distribution of weights – estimation-maximisation algorithm**

(a) 1995 baseline best guesses (Note: this graph is mistakenly labelled “Fellegi Sunter”; it is actually from the estimation-maximisation analysis.

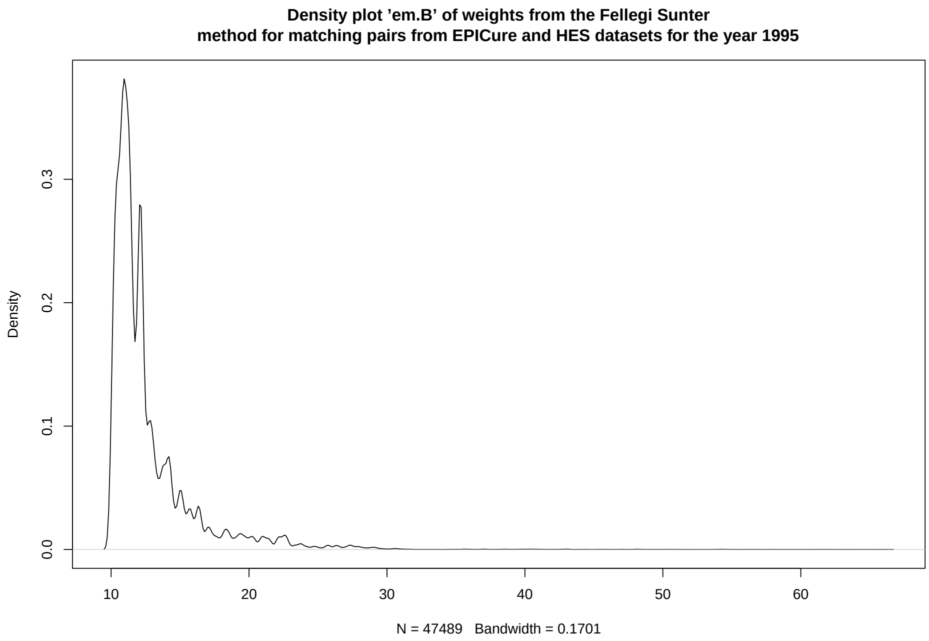

(b) 2006 baseline best guesses

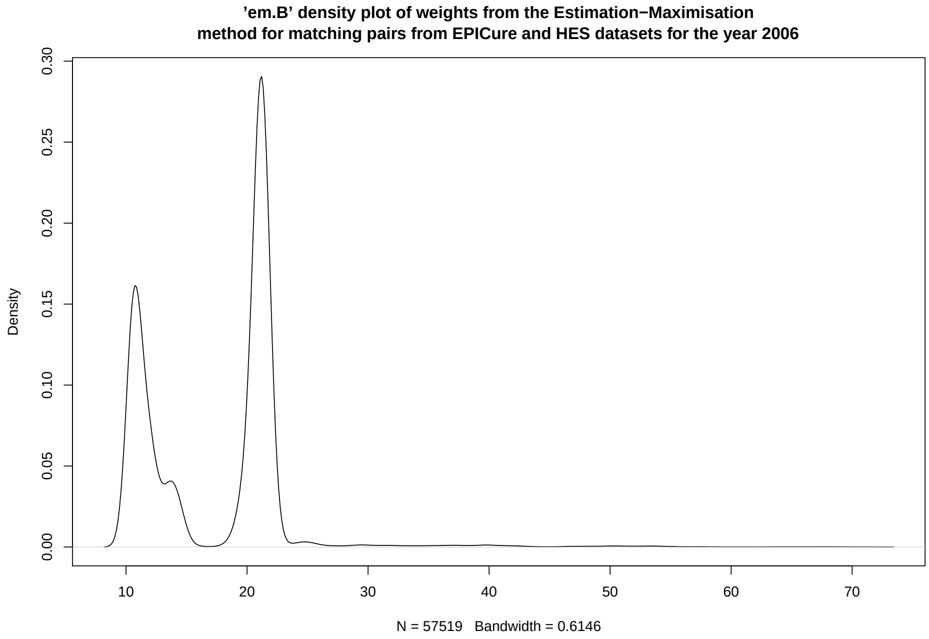

Density distribution of weights from the linkage analyses using the estimation-maximisation algorithm
